# Simultaneous determination of sunset yellow and tartrazine in real samples on an *n*-butylamine-graphite/polyaminophenol composite electrode

**DOI:** 10.55730/1300-0527.3714

**Published:** 2024-12-09

**Authors:** Mürşide Ceren AFŞAR, Zekerya DURSUN

**Affiliations:** Department of Chemistry, Faculty of Science, Ege University, Bornova, İzmir, Turkiye

**Keywords:** Sunset yellow, tartrazine, *n*-butylamine, poly (4-aminophenol), *n*-butylamine intercalated graphite

## Abstract

A composite electrode was fabricated for simultaneous determination of sunset yellow (SY) and tartrazine (Tz) in foods and syrup. It is based on *n*-butylamine intercalation into graphite and then dropped on a poly (4-aminophenol)/glassy carbon electrode (GCE). The electrode surface morphologies, chemistry, and electrical properties were identified by scanning electron microscopy (SEM), X-ray photoelectron spectroscopy (XPS), Raman spectroscopy (RS), and electrochemical impedance spectroscopy (EIS). The new composite electrode significantly increased the electrochemical response of SY and Tz relative to a bare GCE. The peak current of SY and Tz increased linearly depending on their concentrations from 0.006 μmol L^−1^ to 1.0 μmol L^−1^ (r = 0.994) and over two linear ranges from 0.06 to 1.50 μmol L^−1^ (r = 0.9955) and from 2.0 to 10.0 μmol L^−1^ (r = 0.9902), respectively. The limits of detection of SY and Tz were 2.60 nmol L^−1^ and 0.025 μmol L^−1^, respectively (S/N = 3). SY and Tz contents in real samples were determined in syrup, juice powder, and candy. Spiked recovery for samples was from 96.4% to 106.8%. The relative standard deviations were lower than 5.0%.

## 1. Introduction

Synthetic colorants, containing azo dyes, vary in color and are reasonably priced, so they are commonly used to improve the visual grade of beverages and foods. Synthetic azo dyes such as sunset yellow (SY) and tartrazine (Tz) are generally added to foods and drinks such as soft drinks, candies, cakes, and dairy and bakery products [1]. These colorants offer numerous advantages, including high stability towards pH, light, and oxygen; low production costs; and color stability during their shelf life [2]. Because SY and Tz contain azo groups (−N=N−) in their structures, they can be seriously mutagenic and carcinogenic [3]. According to some experiments, these dyes can cause allergies and asthma problems, weight loss of the thymus, diarrhea, hyperactivity disorders in children, and some other symptoms [4]. Because of their potential toxicity to humans, the use of SY and Tz in foods must be regulated by legislation. Therefore, the maximum accepted levels of SY and Tz content added to food and nonalcoholic beverages should be 100 μg mL^−1^ [5] and the limit of daily intake of SY and Tz according to the European Food Safety Authority (EFSA) is defined as a maximum of 4 mg kg^−1^ and 7.5 mg kg^−1^, respectively [6,7]. Because of the variation in the content of SY and Tz in beverages and foods, accurate and precise detection of SY and Tz has become a major interest of analytical chemists. Various spectrophotometric [8], chromatographic [9], and colorimetric [10] methods are currently used in the detection of SY and Tz. However, these methods are time consuming, complex, and costly and require pretreatment steps [11]. Electrochemical methods offer advanced features such as low cost, convenience for detection of various compounds, robustness, high sensitivity, selectivity, reproducibility, short analysis time, and simplicity [12]. To detect azo dye compounds, voltammetric techniques can be utilized by monitoring an electrochemical signal, since their molecular structure contains an azo group (−N=N−) able to undergo reduction and a hydroxyl (OH−) group on the aromatic ring that can undergo oxidation reactions.

In the last two decades, electroanalytical methods have been developed using carbon-type electrodes for the determination of azo dye compounds because of their wide working potential range, low residual current, low cost, easy preparation, and reproducibility. In particular, glassy carbon electrodes (GCEs) [13], graphite [14], graphene [15], and graphene oxide [16] have been used for the determination of SY and Tz. To enhance the analytical properties of voltammetric methods in the detection of SY and Tz, carbon-based electrode materials have been modified by metal particles (Au, Ag) [17,18], oxides [19,20], and conducting polymers [21].

Conducting polymers (CPs) exhibit electronic, magnetic, and optical characteristics and their monomeric units are connected by covalent bonds that are linked by a lengthy conjugation chain. Electrochemical polymerization is a popular method to immobilize the CP on a variety of surfaces. By adjusting the electrochemical parameters, controllable deposition at desired thickness and porosity can be achieved [22].

The existence of CPs in composite electrodes on GCEs provides a physical stable electrode surface in voltammetric measurements [23,24].

Graphite is one of the best known types of layered materials and the distance between its two layers is 3.34 Å [25]. The interlayer spacing allows guest molecules to enter the interlayer region. The interactions between the layers and guest compounds can be detected by precision stabilization between dispersion forces, electrostatic interactions, and Pauli repulsions. As a result of the intercalation of some compounds between these layers, the interaction of graphite with the substances in its environment will improve because of the enhancement in the active surface area of graphite.

Moreover, *n*-butylamine (*n*-BA) is an important industrial feedstock material and by-product of a variety of biological processes [26]. The amine (−NH_2_) group of *n*-BA can easily interact with C atoms in graphite layers via the intercalation process. Therefore, *n*-BA can be used as a constituent of the composite to prepare carbon-based modified electrodes. Compared to different types of conducting polymers, p-aminophenol polymer (PAP) is exceptional in terms of its varied properties. Active functional groups in its structure both allow electrochemical polymerization and establish a selective structure for various compounds [27]. In a recent study, the determination of SY in the presence of Tz was carried out at an overoxidized polyaminophenol modified GCE (Ox-PAP/GCE) [28]. The developed electrode has linear concentration range and good stability, but it has low sensitivity and poor limit of detection (LOD) for SY in the presence of Tz. The sensitivity and selectivity of electroanalytical detection of azo dyes depend on the modifier material properties, such as good conductivity, higher electroactive surface area, a drop in the background current, and improved adsorption capacity.

Graphite-based materials have the advantages of higher conductivity and strong π–π interaction [29], especially after the intercalation of *n*-BA into graphite and then combining with PAP. The composite represents a higher surface area, increased electrochemical activity, and stable adsorption centers for analytes. When compared with pure graphite, in the *n*-BA intercalated graphite–PAP composite, a pair of electrons on nitrogen atoms develops a platform with sp^2^-hybridized carbon frameworks that can enhance the electrochemical response and reactivity of the graphite structure of the composite [30]. Based on this, a novel *n*-BA intercalated graphite–PAP composite electrode was constructed for the simultaneous determination of SY and Tz.

To the best of our knowledge, an *n*-BA intercalated graphite–PAP-based composite electrode has not yet been developed for the simultaneous detection of SY and Tz. The goal of the present study was to fabricate a low cost and highly sensitive and selective composite electrode based on *n*-BA intercalated graphite–PAP/GCE for simultaneous determination of SY and Tz in foods and syrup. Moreover, the surface properties of composite electrode were identified with scanning electron microscopy (SEM), X-ray photoelectron spectroscopy (XPS), Raman spectroscopy (RS), and electrochemical impedance spectroscopy (EIS). Under optimal conditions, we obtained excellent electrochemical results for the simultaneous detection of SY and Tz. Compared to other related studies, this prepared composite electrode is superior in terms of low LOD, wide linear range, high sensitivity, favorable repeatability, and reproducibility. Finally, the detection of SY and Tz in real samples (powdered juice, candy, and syrup) was performed to validate the desired developed composite electrode.

## 2. Materials and methods

### 2.1. Reagents

All the chemicals were of analytical grade. Graphite powder was supplied by Aldrich (1–2 μm, synthetic). Hydrochloric acid (37%), perchloric acid (70%), sulfuric acid (98%), acetic acid (99%), o-phosphoric acid (75%), boric acid ( ≥99.5% ), sodium dodecyl sulfate (SDS) (≥97%), *N,N*-dimethylformamide (DMF) ( ≥99.9% ) (Merck), PAP ( ≥98.0% ) (Fluka), and *n*-butylamine ( 99.5% ) (Sigma-Aldrich) were used. SY and Tz solutions were freshly prepared by dissolving sufficient quantities of them in ultrapure water (Millipore Milli Q system (18.2 MW.cm)). Britton–Robinson (BR) buffer solutions were prepared from 0.04 mol/L acetic acid, 0.04 mol/L phosphoric acid, and 0.04 mol/L boric acid in the pH range from 2.0 to 10.00. The mixture was titrated to the desired pH with 0.2 mol/L NaOH [31]. All experiments were performed under laboratory conditions.

### 2.2. Apparatus

The cyclic voltammetry (CV) and square-wave voltammetric (SWV) experiments were performed using a potentiostat/galvanostat (model PGSTAT-101, Autolab, Utrecht, The Netherlands) controlled by Nova software with a three-electrode system that included a working electrodes as bare GCE (Basi 3 mm diameter and 0.0707 cm^2^ geometric area), polyaminophenol modified GCE (PPAP/GCE), graphite/GCE (G/GCE), an *n*-butylamine/graphite/GCE (*n*-BA/G/GCE), polyaminophenol (PPAP) modified with graphite (G/PPAP/GCE), an *n*-butylamine/graphite/PPAP/GCE (*n*-BA/G/PPAP/GCE), a homemade Ag/AgCl (sat. KCl) reference electrode, and a platinum wire auxiliary electrode.

The pH of the solutions was measured with a WTW handheld 330i ion analyzer. The composite electrode surface properties were identified by SEM (ZEISS GEMINI 500), EIS (Autolab PGSTAT 302N Electrochemical Analyzer), RS (Renishaw Raman spectrometer), and XPS (Thermo K–Alpha–Monochromated high-performance XPS spectrometer).

### 2.3. Fabrication of *n-*BA/G/PPAP/GCE

Prior to covering with composite material, the bare GCE was polished with 0.05 μm alumina on a synthetic cloth and then ultrasonicated in an ethanol/water (1:1 v/v) mixture for 3 min. The PPAP was prepared according to our previous studies [32]. The bare GCE was immersed in a 0.5 mol L^−1^ HClO_4_ containing 1 mmol L^−1^ SDS and 5 mmol L^−1^ PAP monomer solution and then the polymerization of PAP monomer was performed with CV for 10 repetitive potential cycles from −0.5 to 2.0 V (Figure S1) and the obtained surface was denoted as PPAP/GCE. To remove any remaining adsorbed monomer on the surface, it was rinsed with ultrapure water. To generate *n*-BA intercalated graphite suspension, 3 g of graphite and 5 mL of *n*-BA were mixed in an ultrasonic bath for 4 h. The mixture was dried at 50 °C and dispersed in 1 mL of DMF to obtain a black suspension. An aliquot of 10 μL of *n*-BA intercalated graphite suspension was dropped on the GCE or the PPAP/GCE. The solvent on the electrode surfaces was evaporated at 50 °C for 30 min.

### 2.4. Sample preparation

Candy, orange/lemon juice powder (bought from a local market), and syrup (bought from a pharmacy) known to contain SY and Tz were selected as samples. An aliquot of 0.1 mL syrup sample was diluted to 10 mL from BR buffer (pH 4.0) solution. A 5.56-g sugar candy sample was dissolved in 50 mL of BR buffer (pH 4.0). Orange/lemon juice powder (0.015 g and 0.15 g, respectively) was dissolved in 25 mL of BR buffer (pH 4.0).

## 3. Results and discussion

### 3.1. Characterization of electrode surfaces

**Figure 1:**
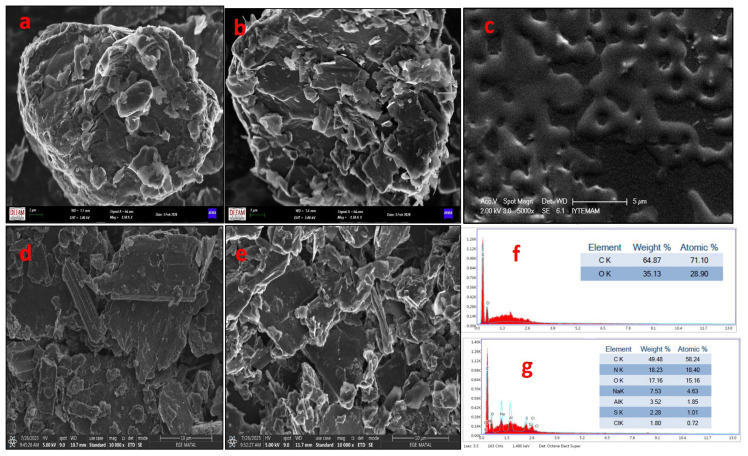
SEM images of (a) graphite 8500× magnitude, (b) *n*-BA intercalated graphite 8500× magnitude, (c) PPAP/GCE 5000× magnitude, (d) G/PPAP/GCE 10,000× magnitude, and (e) *n*-BA/G/PPAP/GCE 10,000× magnitude; EDX results of (f) G/PPAP/GCE and (g) *n*-BA/G/PPAP/GCE.

The SEM micrographs of pure graphite and *n*-BA intercalated graphite are shown in Figure 1. Figure 1a demonstrates a more agglomerate structure of graphite under 8500× magnification. Figure 1b shows *n*-BA intercalated graphite. The SEM micrograph in Figure 1b clearly displays the exfoliation of graphite sheets at 8500× magnification, indicating a close view of separated sheets into thinner layers by *n*-BA intercalation into graphite compared to the structure of pure graphite and that multilayers of graphene could be formed. Figure 1 additionally demonstrates SEM micrographs of the PPAP/GCE, G/PPAP/GCE, and *n*-BA/G/PPAP/GCE. Figure 1c displays the almost homogeneously distributed porous PPAP film on the GCE surface. Figure 1d shows the more agglomerated structure of graphite on the polymer surface under 10,000× magnification. Figure 1e depicts the *n*-BA/G/PPAP/GCE. Figure 1e shows a close-up view of split sheets separated into thinner layers by *n*-BA intercalation into graphite on the polymer surface. To verify the interaction of *n*-BA with graphite, the EDX elemental analyses confirmed the presence of C and O atoms on the G/PPAP/GCE (Figure 1f) and C, O, and N atoms on the *n*-BA/G/PPAP/GCE (Figure 1g). Moreover, the existence of Na, S, and Cl atoms on the *n*-BA/G/PPAP/GCE can be explained by adsorption of supporting electrolyte species during the electrochemical preparation of PAP film on the GCE surface.

**Figure 2:**
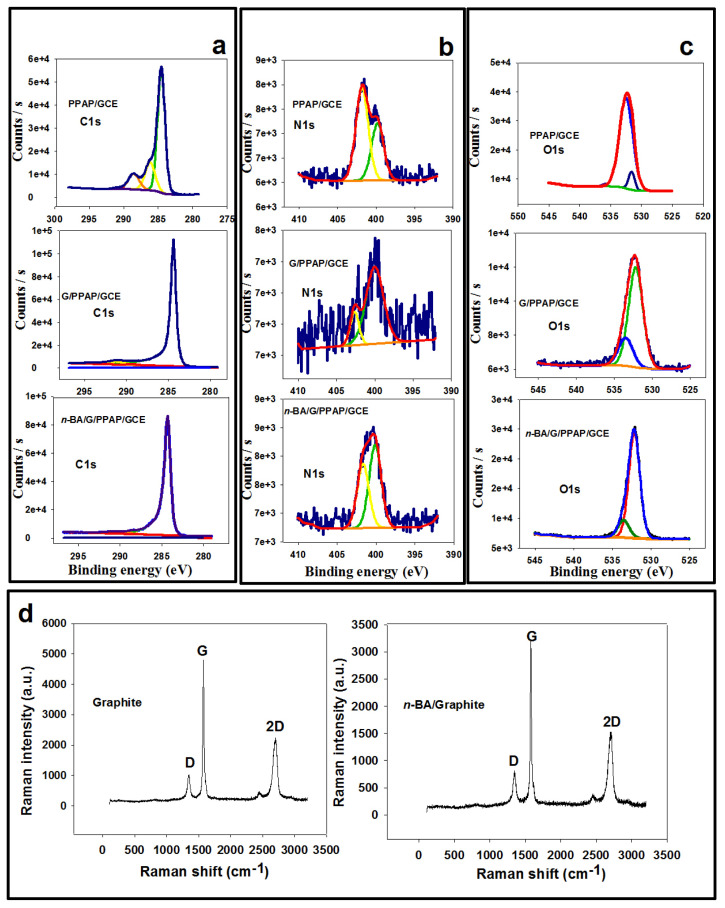
XPS high-resolution spectra of (a) C1s, (b) N1s, (c) O1s of PPAP/GC, G/PPAP/GC, and *n*-BA/G/PPAP/GC electrodes, respectively. (d) RS spectra of graphite and *n*-BA intercalated graphite materials.

The chemical characterization of the PPAP/GC, G/PPAP, and *n*-BA/G/PPAP/GCE surfaces was verified by XPS studies. The XPS results of the modified electrodes are given in Figure 2. Due to the molecular structure of PPAP, G, and *n*-BA/G, the presence of N, C, and O was investigated in the XPS spectrum (Figures 2a–2c). Figure 2a displays the high-resolution XPS spectrum of C1s for the PPAP/GC, G/PPAP, and *n*-BA/G/PPAP modified GCE. The C(1s) spectrum of PPAP/GCE was evaluated at 284.51 eV (sp^2^ hybridized carbon), 286.32 eV (–C–O), and 288.68 eV (C=O). In addition, the C(1s) spectra of G/PPAP/GCE and *n*-BA/G/PPAP/GCE were evaluated at 284.41 eV and 284.36 eV belonging to characteristic sp^2^ hybridized carbon. The N1s spectra of the PPAP/GCE, G/PPAP/GCE, and *n*-BA/G/PPAP/GCE are depicted in Figure 2b. The spectra show two main peaks at 399.9eV and 401.7 eV corresponding to pyrrolic-N (N atom in the pentagonal ring is attached to two adjacent carbons) and graphitic-N (N is attached to three carbon atoms) at the PPAP/GCE. The N(1s) peaks at 402.4 eV and 400 eV corresponded to graphitic-N and pyrrolic-N at the G/PPAP/GC electrode. The N(1s) spectrum of *n*-BA/G/PPAP/GCE was observed at 401.7 eV (graphitic-N) and 400 eV (pyrrolic-N). As expected, N1s binding energy belonging to the graphitic N at the *n*-BA/G/PPAP was considerably higher compared to that of the G/PPAP [33]. The data verify the incorporation of *n*-BA into graphite sheets. The O1s signals of the PPAP, G/PPAP/GCE, and *n*-BA/G/PPAP/GCE are represented in Figure 2c. The curve fits of the O1s peaks for the PPAP/GCE observed at 531.8 eV and 532.6 eV are attributed to the C=O and C–O groups in the polymer structure. Further, the curve fits of the O1s peaks for the G/PPAP/GCE and *n*-BA/G/PPAP/GCE are observed at 532.4 and 533.5, and 532.2 and 533.79 eV, respectively (Figure 2c). The peaks monitored at 532.27 eV and 532.44 eV can be ascribed to the –C=O bond in the polymer chain, while the peaks located at 533.50 eV and 532.79 eV corresponded to the –O–C bond in the polymeric rings [24]. RS provided information about chemical or structural defects in C sp^2^ systems before and after doping procedures. Figure 2d depicts the comparative Raman spectra of graphite and *n*-BA/G. Graphite and *n*-BA/G have sharp G- and 2D-bands, while the D-band belongs to the defected graphite structure. The graphite has a D-band at 1338 cm^−1^, a G-band at 1580 cm^−1^, and a 2D band at 2692 cm^−1^, while for *n*-BA/G, there is a D band at 1331 cm^−1^, a G band at 1564 cm^−1^, and a 2D band at 2684 cm^−1^. The *n*-BA intercalated graphite peak positions of G and 2D were shifted to higher values compared to graphite. The highest blue shifts were 16 cm^−1^ and 8 cm^−1^ for G and 2D peaks, respectively. There are several possible origins for the shift of Raman peaks, indicating an important rise in charge carrier density brought on by the addition of dopant species and strain [34]. Additionally, the peak ratio of the D- and G-bands (I_D_/I_G_) is a critical measure for determining whether a graphite network is orderly or disordered. The *I*_D_/*I*_G_ ratio of graphite was 0.21, while for *n*-BA intercalated graphite it was 0.25. The increase in this value indicates that the moderate intercalation process provides a low defect density in the graphite sheets. In the present study, the I_2D_/I_G_ ratio of the *n*-BA intercalated graphite and pure graphite was 0.48 and 0.45, respectively. Since the I_2D_/I_G_ ratio of *n*-BA intercalated graphite is different from that obtained for pure graphite, multiple graphene layers can be formed by *n*-BA intercalation [35]. Moreover, the Raman spectra were measured from four points of the *n*-BA intercalated graphite. The I_D_/I_G_ ratios for the four points were 0.25, 0.23, 0.36, and 0.20, respectively, demonstrating that the *n*-BA intercalation could be homogeneous into graphite layers.

EIS experiments were performed at all electrodes as specified in order to investigate the rate of electron transfer at all of them (Figure S2a) in the presence of 5.0 mmol L^−1^ [Fe(CN)_6_]^3−/4−^ + 0.1 mol L^−1^ KCl solution at varied frequencies, ranging from 0.50 to 100,000 Hz. In general, all electrodes showed a semicircular Nyquist curve at higher frequencies, demonstrating an electron transfer-limited process, and a linear part at lower frequencies, indicating an electron diffusion-limited process. The obtained data were fitted with an Rs (Cdl(RctW)) equivalent circuit (charge transfer resistance (Rct), electrolyte resistance (Rs), Warburg impedance (W), and the double layer capacitance (Cdl)) and the dataset is given in Table S1. The Rct of the bare GCE has a value of 334 Ω. This indicated a very slow electron transfer rate between the electrode interface and the redox probe. The smallest Rct value (13.9 Ω) of the *n*-BA/G/PPAP/GCE suggests a fast charge transfer compared to the PPAP/GCE (164 Ω), G/GCE (53.7 Ω), G/PPAP/GCE (23.8 Ω), and *n*-BA/G/GCE (16.7 Ω). All of these results indicated that the electron transfer resistance was decreased with the *n*-BA/G/PPAP/GCE. Reversible redox behavior was obtained in the presence of 5.0 mmol L^−1^ [Fe(CN)_6_]^3−/4−^ + 0.1 mol L^−1^ KCl solution at all electrodes (Figure S2b). The best redox behavior was observed for *n*-BA/G/PPAP/GCE.

The electroactive surface areas (EASA) of all electrodes were calculated by the Randles–Sevcik equation (Eq. (1)):


(1) $$\text{Ip}=(2.69\;10^{5})\;{\text{n}}^{3/2}\;\text{A }{\text{D}}^{1/2}\;\text{C }\nu^{1/2},$$

where n is the number of electrons transferred, A is the electroactive surface area (cm^2^), D is the diffusion coefficient (cm^2^ s^−1^), C is the concentration of K_4_Fe(CN)_6_ (mol cm^−3^), and v is the scan rate (V s^−1^). The [Fe(CN)_6_]^3−/4−^ system transfers one electron (n = 1), D was accepted as 6.70 × 10^−6^ cm^2^ s^−1^, and the ferro-ferricyanide concentration (C) was taken 1.0 mmol L^−1^. From the slope of *I_p_* vs. *v^1/2^*, the EASA of the GCE, PPAP/GCE, G/GCE, G/PPAP/GCE, *n*-BA/G/GCE, and *n*-BA/G/PPAP/GCE were obtained as 0.094, 0.115, 0.419, 0.430, 0.432, and 0.515 cm^2^, respectively. As a result, the EASA of the *n*-BA/G/PPAP/GCE was approximately 5.47 times that of the GCE.

### 3.2. Voltammetric behavior of SY and Tz at all electrodes

**Figure 3:**
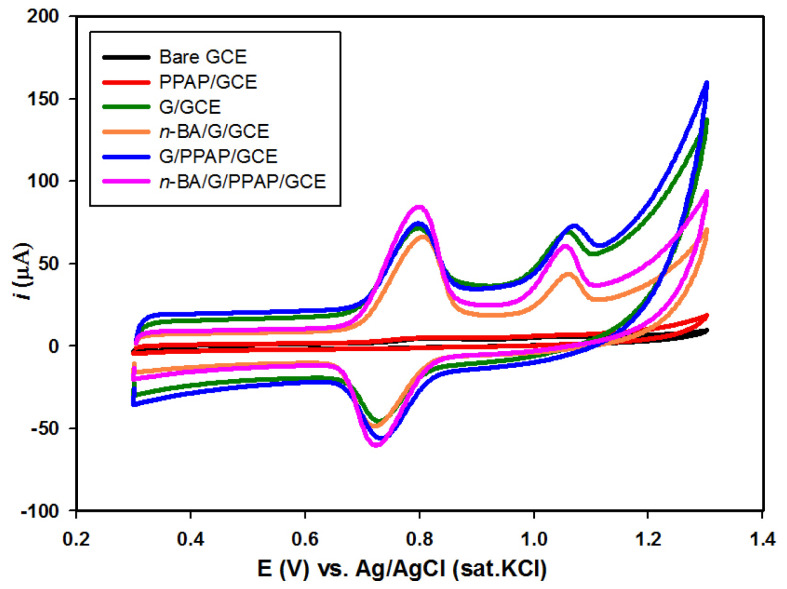
CV responses of bare GCE, PPAP/GCE, G/GCE, *n*-BA/G/GCE, G/PPAP/GCE, and *n*-BA/G/PPAP/GCE in the presence of 1.0 × 10^−4^ mol L^−1^ SY and Tz mixture (1:1) in pH 4.0 BR buffer solution.

The voltammetric behavior of the bare GCE and composite electrodes was examined in the absence and presence of 1.0 × 10^−4^ mol L^−1^ SY and 1.0 × 10^−4^ mol L^−1^ Tz mixture in BR buffer (pH 4.0) solution by CV from 0.30 V to 1.30 V with a 50 mV s^−1^ scan rate (Figure 3). Quasireversible electrochemical behavior was monitored at 0.80 V for oxidation and 0.72 V for the reduction reaction for SY, while irreversible electrochemical behavior was observed at almost 1.05 V for Tz oxidation for all electrodes. Although the oxidation and reduction peaks were located at almost the same potentials for SY at the PPAP/GCE, G/GCE, G/PPAP/GCE, *n*-BA/G/GCE, and *n*-BA/G/PPAP/GCE, the highest peak currents were obtained at the *n*-BA/G/PPAP/GCE (Table S2). Moreover, the oxidation current of Tz was improved at the *n*-BA/G/GCE and *n*-BA/G/PPAP/GCE, while the peak potential was shifted to low positive potentials (1.05 V) compared with the peak located at 1.07 V at the PPAP/GCE, G/GCE, and G/PPAP/GCE. The improved peak characteristics demonstrated electrochemical activity proved by intercalation of *n*-BA into graphite. From the *n*-BA/G containing composite electrodes, the best electrochemical activity was obtained by monitoring the oxidation peak currents at the *n*-BA/G/PPAP/GCE. Therefore, this electrode was preferred for future studies.

### 3.3. Optimization of the *n*-BA/G/PPAP/GCE

To obtain the best conditions for the simultaneous detection of SY and Tz by electrochemical techniques, some main parameters were optimized by varying the PAP monomer concentration, PPAP electropolymerization cycle number, the effect of dispersion of *n*-BA/G powder in DMF at different ratios for the preparation of the *n*-BA/G/PPAP/GC electrode, and solution properties. Figures S3–S5 depict the effect of these parameters on the voltammetric behavior of SY and Tz. The optimal parameters were 5.0 mmol L^−1^ PAP monomer concentration, 10 cycles number of electropolymerization, and 10 μL of *n*-BA (0.03 g of *n*-BA dispersed in 1 mL of DMF).

### 3.4. The pH effect

**Figure 4:**
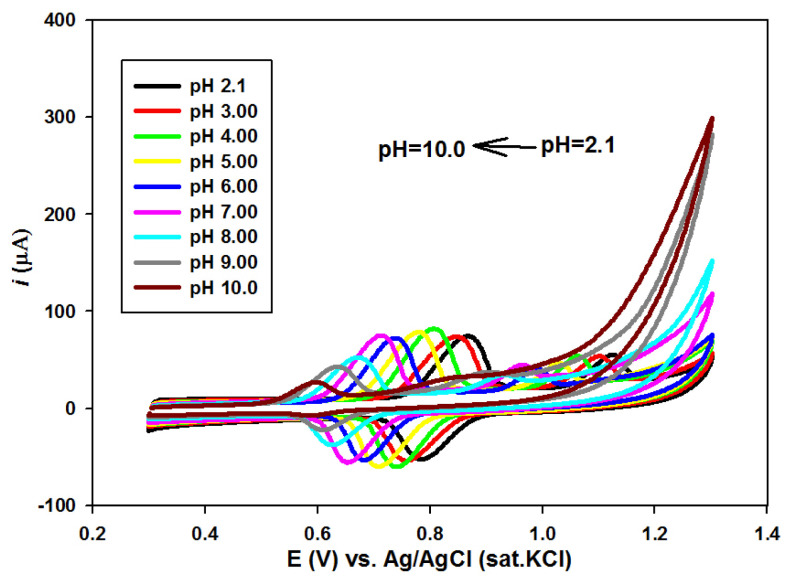
CVs of 1.0 × 10^−4^ mol L^−1^ SY and Tz in the mixture (1:1) at the *n*-BA/G/PPAP/GCE at various pH values (2.1, 3.0, 4.0, 5.0, 6.0, 7.0, 8.0, 9.0, and 10.0) with a scan rate of 50 mV s^−1^.

The pH effect on the simultaneous voltammetric behavior of 1.0 × 10^−4^ mol L^−1^ SY and 1.0 × 10^−4^ mol L^−1^ Tz was examined from 2.1 to 10.0 at the *n*-BA/G/PPAP/GCE with 50 mV s^−1^ (Figure 4). As indicated in Figure 4, the oxidation peak potential (*E_pa_*) and reduction peak potential (*E_pc_*) of SY shifted toward negative values depending on the pH value, while the oxidation peak potentials of Tz were also shifted to negative potentials when pH was increased, indicating that hydrogen ions were involved in both SY and Tz electrode reactions.

**Figure 5:**
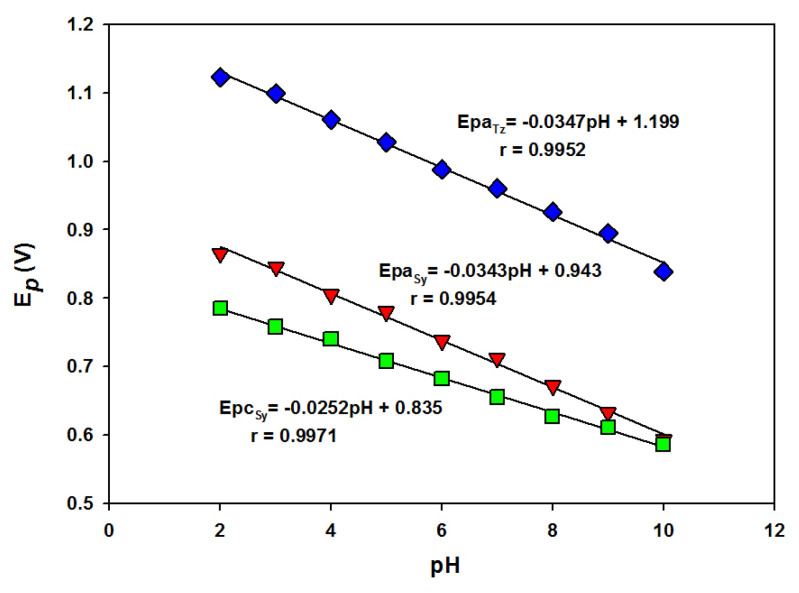
The effects of pH on 1 × 10^−4^ mol L^−1^ SY’s oxidation and reduction and Tz’s oxidation peak potentials.

In Figure 5, it is seen that there was a linear change between peak potentials and pH. The linear equations for both oxidation and reduction peak potentials of SY were *Epa_SY_* = −0.0343pH + 0.9439 (r = 0.9954) and *Epc_SY_* = −0.0252pH + 0.835 (r = 0.9971) and the linear equation for oxidation peak potential of Tz was *Epa_Tz_* = −0.0347pH + 1.1996 (r = 0.9952) (Figure 5). The slopes of −0.0343 V pH^−1^ and −0.0347 V pH^−1^ demonstrated that the ratio of electrons and hydrogen ions number taking part in the electrode reaction was 2:1 for both SY and Tz [36]. In addition, the higher peak currents of SY and Tz were obtained in BR buffer (pH 4.0). Therefore, BR buffer (pH 4.0) solution was chosen for future experiments.

### 3.5. Scan rate effect

**Figure 6:**
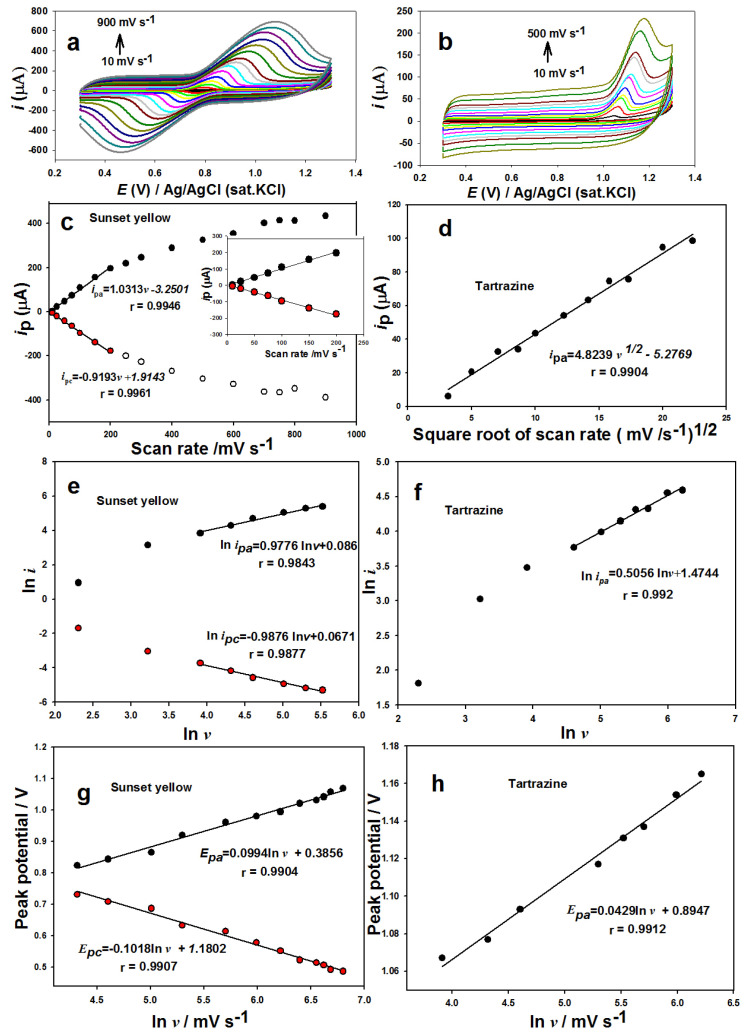
CVs of SY (a) and Tz (b) at different scan rates. The plots of (c) the redox peak currents versus scan rate (*v*) for SY and (d) oxidation current versus the square root of scan rate (*v*^1/2^) for Tz. The relationship between ln i and the ln *v* for (e) SY and (f) Tz. The relationships between peak potential and ln *v* for (g) SY and (h) Tz.

The scan rate effect on the voltammetric behavior of both 1.0 × 10^−4^ mol L^−1^ SY and 1.0 × 10^−4^ mol L^−1^ Tz was studied by CV in the range of 10 to 900 mV s^−1^ and 10 to 500 mV s^−1^ at the *n*-BA/G/PPAP/GCE, respectively (Figures 6a and 6b). The peak potentials of SY shifted slightly towards positive and negative values, while the oxidation peak potential of Tz shifted to negative values, depending on scan rates, indicating that a quasireversible electrode process for SY and an irreversible electrode process for Tz were performed at the *n*-BA/G/PPAP/GCE. In Figure 6c, the redox peak heights of SY increased linearly with the scan rate (*v*) in the range from 10 to 200 mV s^−1^ and the equations were obtained as *i*_pa_ (μA) = 1.0313 *v* (mV s^−1^) −3.2501 (r = 0.9946) and *i_pc_* (μA) = −0.9193 *v* (mV s^−1^) + 1.9143 (r = 0.9961), respectively. The obtained slope’s value was close to 1.0. Therefore, the electrode reaction could be controlled by adsorption processes for SY. Moreover, in Figure 6d, the peak current of Tz (*i_p_*) was linear depending on the square root of scan rate (ν^1/2^) in the range from 10 to 200 mV s^−1^ and the equation can be expressed as *i*_pa_ (μA) = 4.8239 *v*^1/2^ (mV s^−1^)^1/2^ −5.2769 (r = 0.9904), indicating that the electrode process could be controlled by diffusion at the *n*-BA/G/PPAP/GCE. The linear relationships between ln *i* and ln *v* are depicted in Figure 6e and Figure 6f and the equations can be expressed as follows: ln *i*_pa_ (μA) = + 0.9776ln *v* (mV s^−1^) + 0.086 (r = 0.9843) and ln *i*_pc_ (μA) = −0.9876 ln *v* (mV s^−1^) + 0.0671 (r = 0.9877) for SY and ln *i*_pa_ (μA) = 0.5056 ln *v* (mV s^−1^) + 1.4744 (r = 0.992) for Tz. Since the slope values of 0.9776 and 0.9876 were close to 1.0, the electrode reaction of SY was also adsorption controlled, while the 0.5056 slope value for Tz corresponds to a diffusion-controlled electrode reaction. The function Ep = f (ln (υ)) was examined to calculate the quantity of electrons transferred during the redox processes of SY (Figures 6g and 6h). The slopes of the curves for E_pa_ and E_pc_ can be represented using Laviron’s model [37]:


(2) $${\text{E}}_{\text{pa}}=E^{0^{\prime }}+RT/(1-\alpha )nFln\;v$$

(3) $$E_{pc}=E^{0^{\prime }}-RT/\alpha nFlnv$$

*E^0′^* is the formal potential (V), *E_pa_* is the peak potential (V), *R* is the universal gas constant (8.314 J K^−1^ mol^−1^), *α* is the charge transfer coefficient, *T* is the temperature (K), *F* is the Faraday constant (96.485 C mol^−1^), and *n* is the number of electrons transferred in the electrode process. The *α* can be calculated as 0.49 from Eq. (2) and Eq. (3) for SY. According to Laviron’s research on adsorption-controlled currents for reversible processes, the equation is (Ep – Ep_1/2_) = 45/((1 – α) × n) [38]. The *n* can be calculated as 1.56 (approximately equal to 2). SY has a reversible two-electron one-proton reaction. In terms of an irreversible process, *E_pa_* is described by Laviron’s equation [35], Eq. (4):


(4) $$E_{pa}=E^{0^{\prime }}+\frac{RT}{\alpha nF}lnv$$

The slope of the *E_pa_–lnv* line is denoted by *RT/αnF*. *E_pa_* is shifted positively by 1.15RT/αF (or 30/α mV at 25 °C) for every ten-fold increase in *v* [39]. The α can be determined as 0.36. Number of electrons transferred (n) can be determined as 1.65 (approximately equal to 2). As a result, Tz oxidation has an irreversible two-electron one-proton reaction.

### 3.6. SWV detection of SY and Tz

The CV data indicate that the peaks of SY and Tz in their mixture were separated at all electrodes, while higher peak currents were observed for both SY and Tz at the *n*-BA/G/PPAP/GCE. SWV was used for selective simultaneous detection of SY and Tz. For this aim, the effects of SWV parameters, such as amplitude and frequencies, on the peak currents were monitored for 1.0 μmol L^−1^ SY and 5.0 μmol L^−1^ Tz in BR buffer (pH 4.0). The optimum SWV parameters were 2.0 Hz and 0.02 V for frequency and amplitude, respectively. Since the electrode reaction of SY was adsorption controlled, the accumulation time and SY containing BR buffer solution stirring by magnetic bar were also optimized by monitoring of oxidation peak current. An accumulation time of 240 s and a stirring rate of 900 rpm were found for 1.0 μmol L^−1^ SY in the presence of 5.0 μmol L^−1^ Tz. Under optimal conditions, the detection of SY and Tz by SWV was performed with two different methods. In the first method, only the concentration of one compound was varied, while the concentration of the other analyte was kept constant; in the second method, the SY and Tz concentrations were simultaneously varied.

**Figure 7:**
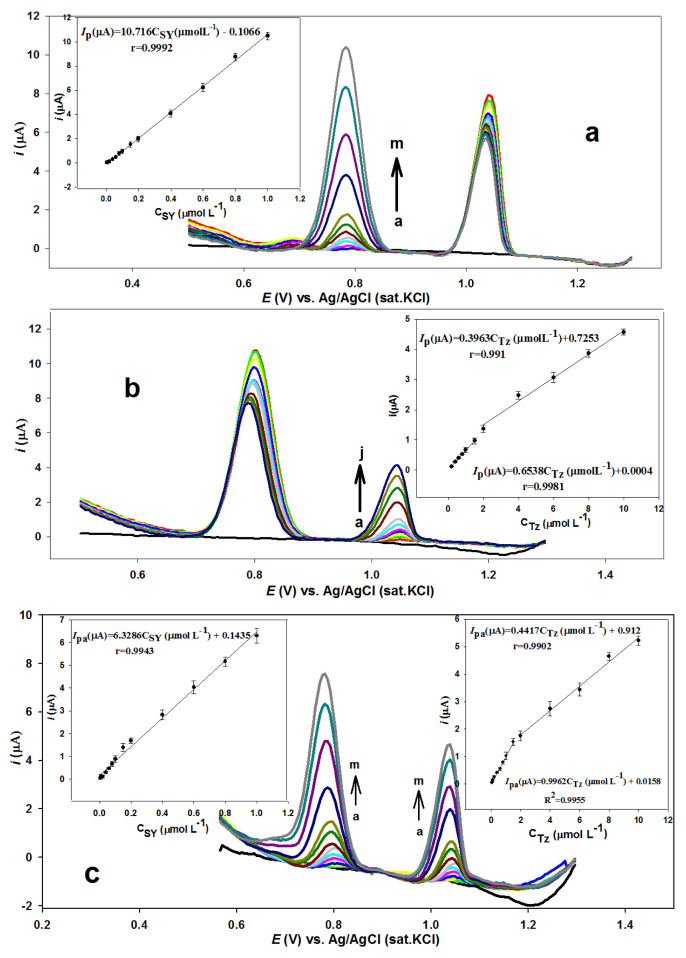
(a) SWV of different concentrations of SY (a–m: 0.006–1.0 μmol L^−1^) in the presence of 10.0 μmol L^−1^ Tz. (b) SWV of various concentrations of Tz (a–j: 0.2–10.0 μmol L^−1^) in the presence of 1.0 μmol L^−1^ SY. (c) SWV of various concentrations of SY (a–m: 0.006–1.0 μmol L^−1^) and Tz (a–m: 0.06–10.0 μmol L^−1^). Inset: the plots demonstrating the oxidation peak current against various SY and Tz concentrations.

As shown in Figure 7a, the SWV results were obtained by increasing the SY concentration from 0.006 μmol L^−1^ to 1.0 μmol L^−1^ in the presence of 10.0 μmol L^−1^ Tz. The peak current of SY monitored at 0.80 V was increased by increasing its concentration, while the oxidation peak current of Tz monitored at 1.04 V was not significantly changed. The drop in the peak current of Tz may be ascribed to the fact that SY is the first to be oxidized and reduces the interaction area of the electrode. The correlation between peak current and SY concentration was determined as linear with the regression equation *i*_p_(μA) = 10.716C_SY_ (μmol L^−1^) – 0.1066 (r = 0.9992). The LOD was calculated with the equation LOD = 3.3σ/m, where is the σ is the standard deviation of the response of buffer solution and m is the slope of the calibration curve. LOD was calculated as 2.32 nmol L^−1^. Similar experiments were performed by increasing the Tz concentration from 0.20 μmol L^−1^ to 10 μmol L^−1^ in the presence of 1.0 μmol L^−1^ SY (Figure 7b). The peak current of Tz formed at 1.04 V was increased by increasing its concentration, while a small change was observed in the peak current of SY at 0.80 V. The calibration graph for Tz has two linear segments; the first segment increases from 0.20 μmol L^−1^ to 1.5 μmol L^−1^ with a linear regression equation of *i*_p_(μA) = 0.6538C_Tz_ (μmol L^−1^) + 0.0004 (r = 0.9981) and the second segment increases from 2.0 μmol L^−1^ to 10.0 μmol L^−1^ with *i*_p_ (μA) = 0.3963C_Tz_ (μmol L^−1^) + 0.7253 (r = 0.991) (Figure 7b). The LOD calculated for Tz was 0.06 μmol L^−1^. Moreover, the simultaneous detection of SY and Tz was also investigated by increasing of both analytes’ concentrations at the *n*-BA/G/PPAP/GCE. As represented in Figure 7c, the peak current of SY was increased linearly between 0.006 μmol L^−1^ and 1.0 μmol L^−1^ with the linear regression equation *i*_p_ (μA) = 6.3286C_SY_ (μmol L^−1^) + 0.1435 (r = 0.9943). The LOD was 2.60 nmol L^−1^. Moreover, the peak current of Tz was also increased linearly between 0.06 μmol L^−1^ and 10.00 μmol L^−1^. The calibration graph has two linear segments from 0.06 μmol L^−1^ to 1.50 μmol L^−1^ with a linear regression equation of *i*_p_(μA) = 0.9962C_Tz_ (μmol L^−1^) + 0.0158 (r = 0.9955) and from 2.0 μmol L^−1^ to 10.0 μmol L^−1^ with *i*_p_(μA) = 0.4417C_Tz_ (μmol L^−1^) + 0.912 (r = 0.9902) at the *n*-BA/G/PPAP/GCE. The LOD was 0.025 μmol L^−1^ for Tz (S/N = 3).

Table 1 shows the comparison of the fabricated electrode with the previously published papers for simultaneous detection of SY and Tz, which demonstrated the highest sensitivity and lowest LOD of the simple and low cost fabricated composite electrode, indicating the advantages of this composite.

### 3.7. Interferences

To estimate the selectivity of the *n*-BA/G/PPAP/GCE and SWV for the simultaneous determination of SY and Tz, possible interferences studies of inorganic and organic compounds were performed under optimized conditions. A relative error of ±10% was regarded as acceptable. The influence of inorganic and organic compounds at several molar ratios over 1.0 μmol L^−1^ SY and Tz were monitored on the oxidation peak currents, and the results are given in Table S3. As shown, the peak currents of SY and Tz were not significantly varied in the presence of K^+^, Na^+^, Ca^2+^, Zn^2+^, Mg^2+^, Cl^−^, Ac^−^, NO_3_^−^, SO_4_^2−^, L-ascorbic acid, glucose, citric acid, or oxalic acid until 1000-fold excess amount of them. However, interference was obtained for SY and Tz peak current in the presence of one-fold and more than one-fold of Allura Red, Brilliant Blue, and Quinoline Yellow. From these results, the developed *n*-BA/G/PPAP/GCE can be used to successfully determine SY and Tz contents in real samples.

### 3.8. Analysis of SY and Tz in real samples

In order to estimate the validity of the developed SWV method, the content of SY and Tz in syrup, juice powder (lemon), juice powder (orange), and candy were analyzed under optimum conditions at the *n*-BA/G/PPAP/GCE. The samples were supplied by local markets and each sample was prepared as described in section 2.4. The standard addition method was used. To analyze and improve the accuracy, the results were obtained three times. The results are summarized in Table 2. The recovery range of the spiked samples was from 96.4% to 106.8% and the RSD was less than 5%, indicating that the developed electrode can be applied for individual or simultaneous detection of SY and Tz.

### 3.9. Reproducibility, repeatability, and stability of the n-BA/G/PPAP/GCE

The reproducibility of the *n*-BA/G/PPAP/GCE response for 0.02 μmol L^−1^ and 0.2 μmol L^−1^ concentrations of SY and Tz mixture was monitored with ten different electrodes. The RSDs of the analytes’ peak currents were 4.64% for SY and 3.17% for Tz; the data confirm that good response reproducibility was obtained for the developed electrode. The repeatability response of the *n*-BA/G/PPAP/GCE was considered by five repetitive measurements. The RSD was calculated as 0.98% for SY and 1.72% for Tz. The results indicate that the *n*-BA/G/PPAP/GCE has good reproducibility and repeatability. Moreover, the peak currents of 0.2 μmol L^−1^ SY and 0.2 μmol L^−1^ Tz containing BR buffer (pH 4.0) were followed to estimate the stability of the composite electrode. To estimate the stability of the *n*-BA/G/PPAP/GCE, it was used after being protected in the space of the supporting electrolyte at room temperature. After 3 days, the peak currents of analytes at the electrode were 83.41% and 80.85% of their initial currents for SY and Tz, respectively.

## 4. Conclusion

A novel composite electrode was successfully developed by intercalation of *n*-BA into graphite layers and then incorporated with PPAP on the GCE surface. The surface chemistry, morphology, and electrical properties of the *n*-BA/G/PPAP/GCE were identified using XPS, FTIR, RS, SEM, and EIS. In particular, the differences in peak shifts in graphite and *n*-BA intercalated graphite in RS studies indicated that a composite structure consisting of multilayer graphene could be formed during *n*-BA’s intercalation into graphite. The composite electrode shows good charge transfer and high electrochemical activity for simultaneous detection of SY and Tz. The composite electrode demonstrated satisfactory analytical characteristics such as wide linear concentration range, low detection limit, good stability, and high reproducibility, selectivity, and sensitivity towards both SY and Tz. Successful real sample analyses were performed at the developed composite electrode in four different types of samples. Finally, the sensitivity experiment results obtained with the developed composite electrode showed that lower amounts of SY and Tz can be determined to compare with the daily intake limit amount of SY and Tz according to the EFSA.

## Supplementary materials

Figure S1 Cyclic voltammograms of PAP polymerization on GCE in 0.5 mol L^−1^ HClO_4_ containing 1.0 mmol L^−1^ SDS and 5.0 mmol L^−1^ p-aminophenol from 1th cycle to 10^th^ cycle.

Figure S2 (a) Cyclic voltammograms of Bare, PPAP/GCE, G/GCE, *n*-BA/G/GCE, G/PPAP/GCE and *n*-BA/G/PPAP/GCE in a 0.1 mol L^−1^ KCl solution containing 5.0 mmolL^−1^ [Fe(CN)_6_]^3−/4−^ at scan rate of 50 mVs^−1^. (b) The Nyquist plots of the bare and modified electrodes in the 5.0 mmol L^−1^ K_3_[Fe(CN)_6_]/K_4_[Fe(CN)_6_] containing 0.1 mol L^−1^ KCl solution at varying frequencies range 0.5 to 100.000 Hz at the formal potential. Inset is the Randles circuit model for the modified electrodes.

Figure S3 The effect of cycle number on analyte peak currents in PPAP electropolymerization

Figure S4 The effect of PPAP concentration on analyte peak currents in PPAP electropolymerization

Figure S5 The cyclic voltammetric behavior of 1.0x10^−4^ mol L^−1^ SY and Tz prepared in different amounts of *n*-BA/G/PPAP/GC electrodes and related current quantity graph

**Table S1:** Data obtained after fitting the electrochemical impedance spectra with the equivalent circuit.

	R (Ω)	Cdl (μF)	Rct (Ω)	W (mMho)
**Bare GCE**	172	1.11	334	1.26
**PPAP/GCE**	122	20.6	164	1.39
**G/GCE**	160	5.99	53.7	1.77
**G/PPAP/GCE**	779	9.62	23.8	6.63
***n*** **-BA/G/GCE**	72.6	4.92	16.7	3.83
***n*** **-BA/G/PPAP/GCE**	75.3	1.54	13.9	6.58

**Table S2:** Peak current and potential of 0.1 mmol L^−1^ SY and Tz at modified electrodes

Electrode	SY (0.1 mmol L ^−1^) E(V)	i(μA)	Tz (0.1 mmol L ^−1^) E(V)	i(μA)
**Bare GCE**	0.805	1.78	1.070	0.704
**PPAP GCE**	0.791	1.38	1.072	0.148
**G/GCE**	0.793	43.79	1.057	19.40
***n*** **-BA/G/GCE**	0.803	51.65	1.057	18.79
**G/PPAP/GCE**	0.797	45.34	1.063	19.92
***n*** **-BA/G/PPAP/GCE**	0.797	66.09	1.053	27.88

**Table S3:** Effect of coexisting ions 1.0 μmol L^−1^ SY and Tz in the mixed solution (1:1)

Interferences	Molar ratio ([Interfering species]/[SY+Tz])	Change on the analytical signal of SY (%)	Change on the analytical signal of Tz (%)
Na ^+^	1	−0.4	0.1
	10	1.1	1
	100	0.8	1
	1000	−7.9	−7.7
K ^+^	1	3	3.9
	10	4.6	1.4
	100	3.9	0.6
	1000	−5	−4.7
Zn ^2+^	1	−1.8	2.2
	10	4.1	−0.4
	100	3.8	0.3
	1000	−3.9	−5.7
Ca ^2+^	1	−2.2	0.1
	10	−3	−0.3
	100	−3.1	3.1
	1000	−4	−5
Cl ^−^	1	−0.4	−0.7
	10	−0.3	−0.7
	100	−0.4	−0.7
	1000	−6.9	−1.4
SO _4_ ^2−^	1	3	3.9
	10	4.6	1.4
	100	3.9	0.6
	1000	−5	−4.7
NO _3_ ^−^	1	−2.2	0.1
	10	−3	−0.3
	100	−3.1	3.1
	1000	−4	−5
CH _3_COO ^−^	1	−0.4	0.1
	10	1.1	1
	100	0.8	1
	1000	−7.9	−7.7
Mg ^2+^	1	−0.4	−0.7
	10	−0.3	−0.7
	100	−0.4	−0.7
	1000	−6.9	−1.4
Glucose	1	0.2	−0.5
	10	2.7	0.1
	100	3.8	−0.2
	1000	−0.2	−1.3
Ascorbic asid	1	1.4	−0.5
	10	2.9	0.1
	100	3.1	−0.2
	1000	−6	−4.8
Citric asid	1	0.3	0.6
	10	1	0.6
	100	1.8	−1
	1000	−5	−5
Oxalic acid	1	1.7	−0.2
	10	2.1	0.8
	100	2.8	3.9
	1000	−6.5	−
Allura Red	1	−25	−42.7
	10	-	−84
	100	-	-
	1000	-	-
Brillant Blue	1	−7.5	−39.9
	10	−36.7	-
	100	-	-
	1000	-	-
Quinoline Yellow	1	−22.5	−60.3
	10	−76.3	-
	100	-	-
	1000	-	-

## Figures and Tables

**Table 1:** Comparison of different modified electrodes for SY and Tz determination.

Electrode	Methods	Supporting electrolyte	SY Linear range μmol L^−1^	LOD μmol L^−1^	Tz Linear range μmol L^−1^	LOD μmol L^−1^	Sample	Ref.
FL-MoS_2_/SPE	DPV	0.1 M PBS (pH 7.0)	0.1–150.0	0.04	-	-	Apple/orange juice	[2]
nAu-CPE	DPV	0.1 M PBS (pH 4.0)	0.1–2.0	0.03	0.05–1.6	0.009	Orange juice, fruit juice	[4]
In^3+^/NiO RLHNSs/GCE	DPV	0.1 M PBS (pH 5.0)	0.01–700.0	0.0027	0.01–700.0	0.0031	Jelly, fruit juice	[5]
Ppy-SCNT-COOH/GCE	SWV	BR buffer (pH 7.0)	0.005–1.0	0.0007	0.007–1.0	0.001	Jelly, candy, ice cream, juice powder, soft drink	[11]
ZnO/Cysteic acid/GCE	DPV	0.1 M PBS (pH 5.0)	0.1–3	0.031	0.07–1.86	0.01	Orange and fruit juice, peach gelatin	[12]
Co_C_-CPE	SWV	0.001 M PBS (pH 3.1)	0.12–3.0	0.9	0.12–3.0	0.3	orange and strawberry flavored gelatin, powdered, soft drinks	[40]
Fe_2_Zr_2_O_7_/GCE	DPV	BR buffer (pH 2.0)	19–270	5.7	19–270	13	soft drinks	[41]
*n*-BA/G/PPAP/GCE	SWV	BR buffer (pH 4.0)	0.006–1.0	0.0026	0.06–10.0	0.025	Syrup, Juice powder, candy	This work

FL-MoS_2_/SPE: Flower-like MoS2 screen printed electrode; DPV: differential pulse voltammetry; nAu-CPE: gold nanoparticles carbon paste electrode; In^3+^/NiO RLHNSs/GCE: In^3+^/NiO hierarchical nanostructures modified glassy carbon electrode; Ppy-SCNT-COOH/GCE: polypyrrole (ppy) decorated oxidized single-walled carbon nanotubes modified glassy carbon electrode; SWV: square-wave voltammetry; ZnO/Cysteic acid/GCE: ZnO/Cysteic acid modified glassy carbon electrode; Co_C_-CPE: cobalt complex carbon paste electrode; Fe_2_Zr_2_O_7_/GCE: iron-zirconium binary transition metal oxide modified glassy carbon electrode; *n*-BA/G/PPAP/GCE: *n*-Butylamine intercalated graphite poly (4-aminophenol) film glassy carbon electrode

**Table 2:** The detection of SY and Tz contents in four different types of samples (n = 3).

Sample	Synthetic dyes	Added (mol/L)	Expected (mol/L)	Found (mol/L)	Recovery (%)	RSD (%)
**Syrup**	SY	0	–	4.70×10^−9^	–	4.36
		2×10^−8^	2.47×10^−8^	2.39×10^−8^	96.8	2.55
		4×10^−8^	4.47×10^−8^	4.59×10^−8^	102.8	3.49
		6×10^−8^	6.47×10^−8^	6.63×10^−8^	102.5	3.70
**Juice powder (lemon)**	Tz	0	–	7.44×10^−8^	–	2.80
		10^−7^	1.74×10^−7^	1.80×10^−7^	103.4	0.34
		2×10^−7^	2.74×10^−7^	2.83×10^−7^	103.1	1.54
		4×10^−7^	4.74×10^−7^	4.94×10^−7^	104.3	3.08
**Juice powder (orange)**	SY	0	–	9.50×10^−9^	–	4.56
		2×10^−8^	2.95×10^−8^	3×10^−8^	101.6	4.62
		4×10^−8^	4.95×10^−8^	4.89×10^−8^	98,9	1.86
		6×10^−8^	6.95 ×10^−8^	7.20×10^−8^	103.5	4.80
	Tz	0	–	1.13×10^−7^	–	4.14
		2×10^−7^	3.13×10^−7^	3.25×10^−7^	104.5	3.06
		4×10^−7^	5.13×10^−7^	5.48×10^−7^	106.8	4.28
		6×10^−7^	7.13×10^−7^	7.37×10^−7^	103.3	4.18
**Candy**	SY	0	–	4.40×10^−8^	–	2.73
		10^−7^	1.44×10^−7^	1.38×10^−7^	96.4	3.21
		2×10^−7^	2.44×10^−7^	2.45×10^−7^	100.5	3.78
		3×10^−7^	3.44×10^−7^	3.40×10^−7^	98.8	4.31
	Tz	0	–	4.20×10^−7^	–	2.92
		10^−6^	1.42×10^−6^	1.51×10^−6^	106.3	3.01
		2×10^−6^	2.42×10^−6^	2.43×10^−6^	100.4	3.80
		3×10^−6^	3.42×10^−6^	3.37×10^−6^	98.0	1.58
