# Supplementary figures and images for: Simultaneous determination of sunset yellow and tartrazine in real samples on an n-butylamine-graphite/polyaminophenol composite electrode
Source: Turk J Chem. 2024 Dec 9;49(1):103–17. doi: 10.55730/1300-0527.3714 (PMC11913369; doi:10.55730/1300-0527.3714)

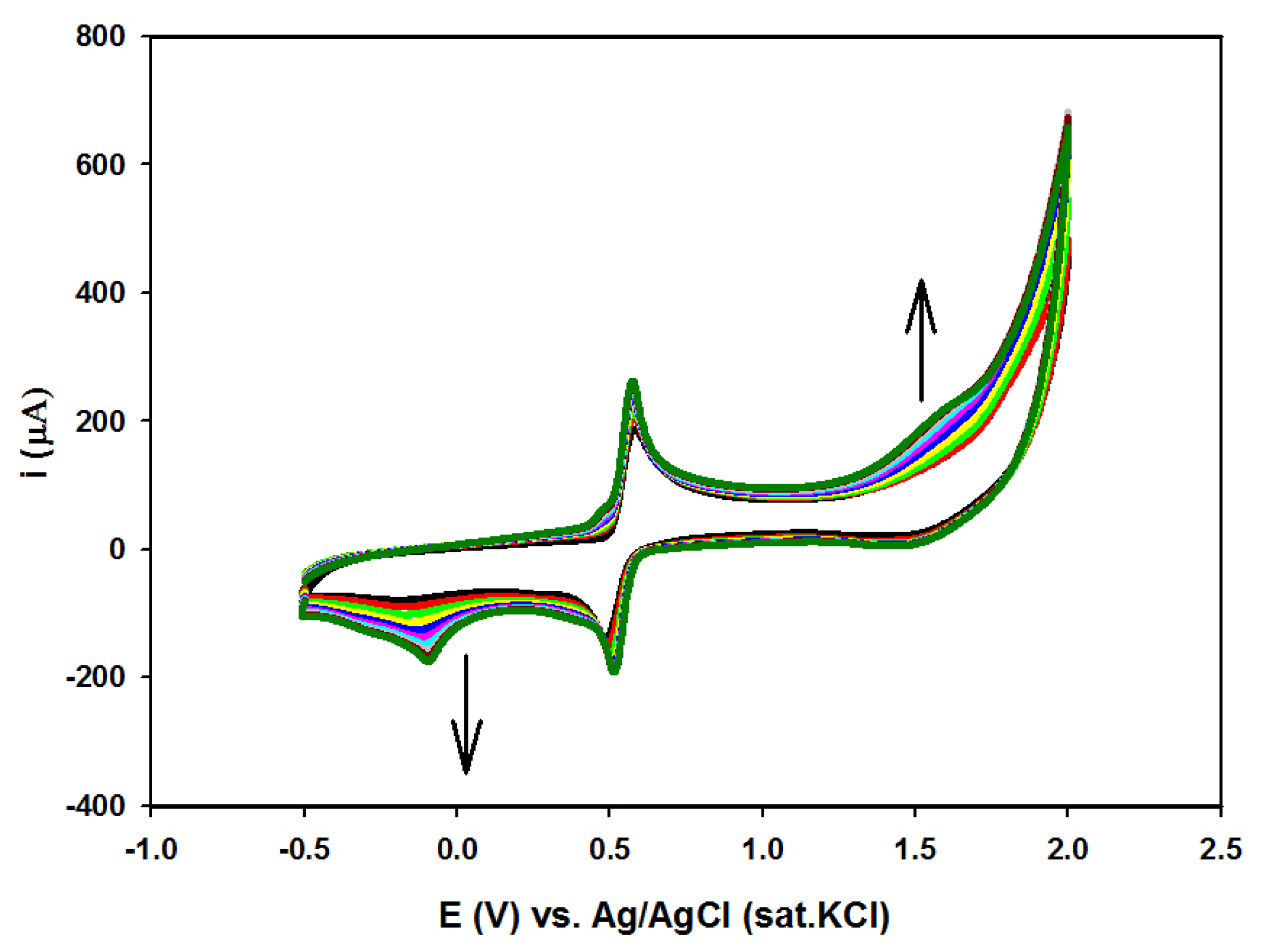

Supplement: Figure S1 — Cyclic voltammograms of PAP polymerization on GCE in 0.5 mol L−1 HClO4 containing 1.0 mmol L−1 SDS and 5.0 mmol L−1 p-aminophenol from 1th cycle to 10th cycle. [file tjc-49-01-103s1.tif]

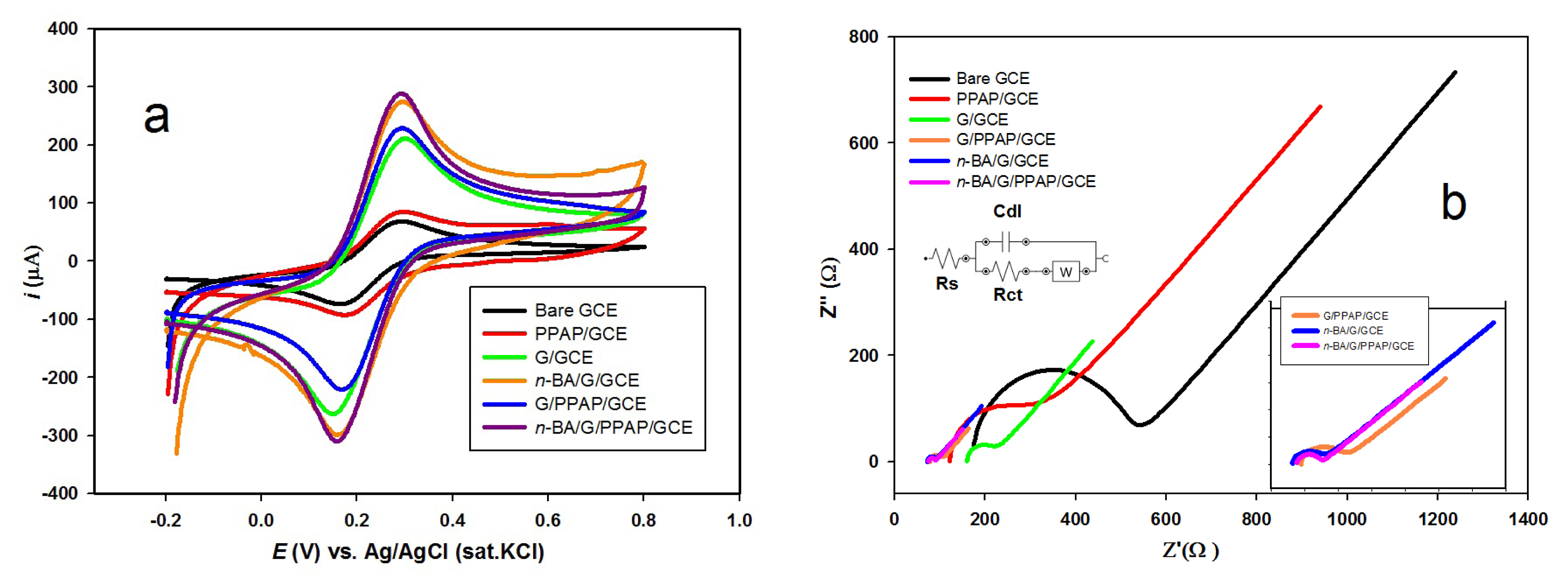

Supplement: Figure S2 — (a) Cyclic voltammograms of Bare, PPAP/GCE, G/GCE, n-BA/G/GCE, G/PPAP/GCE and n-BA/G/PPAP/GCE in a 0.1 mol L−1 KCl solution containing 5.0 mmolL−1 [Fe(CN)6]3−/4− at scan rate of 50 mVs−1. (b) The Nyquist plots of the bare and modified electrodes in the 5.0 mmol L−1 K3[Fe(CN)6]/K4[Fe(CN)6] containing 0.1 mol L−1 KCl solution at varying frequencies range 0.5 to 100.000 Hz at the formal potential. Inset is the Randles circuit model for the modified electrodes. [file tjc-49-01-103s2.tif]

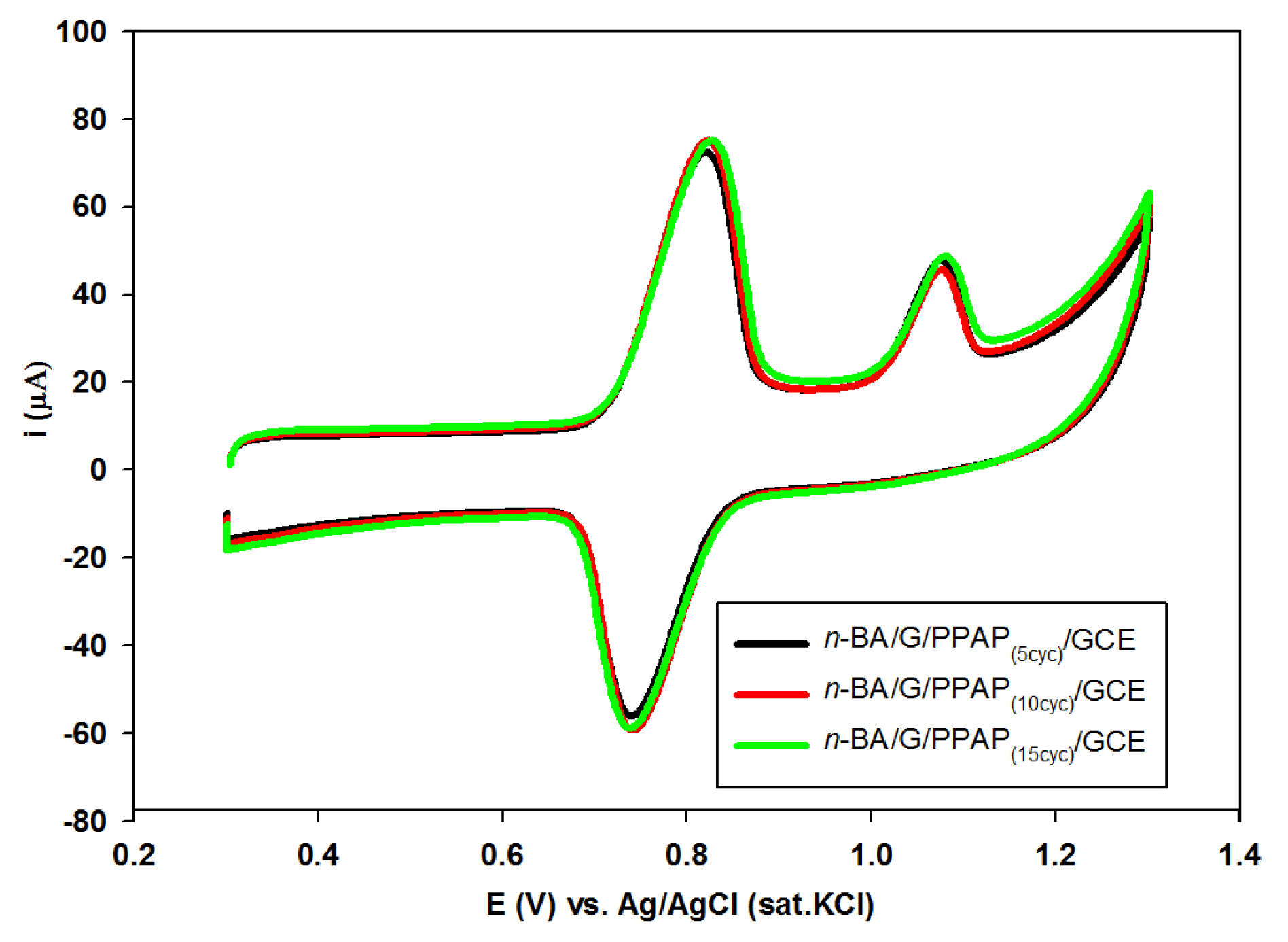

Supplement: Figure S3 — The effect of cycle number on analyte peak currents in PPAP electropolymerization [file tjc-49-01-103s3.tif]

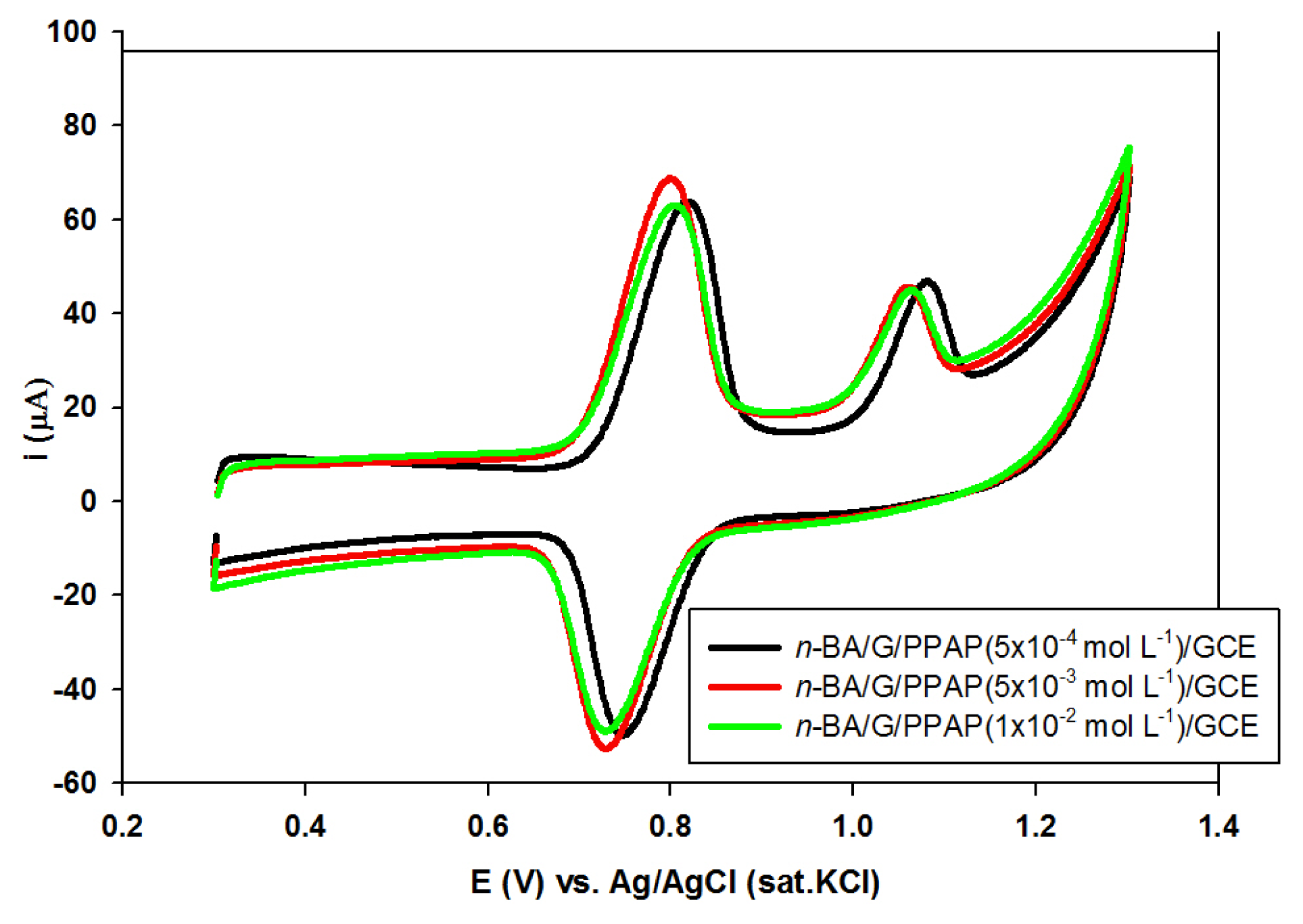

Supplement: Figure S4 — The effect of PPAP concentration on analyte peak currents in PPAP electropolymerization [file tjc-49-01-103s4.tif]

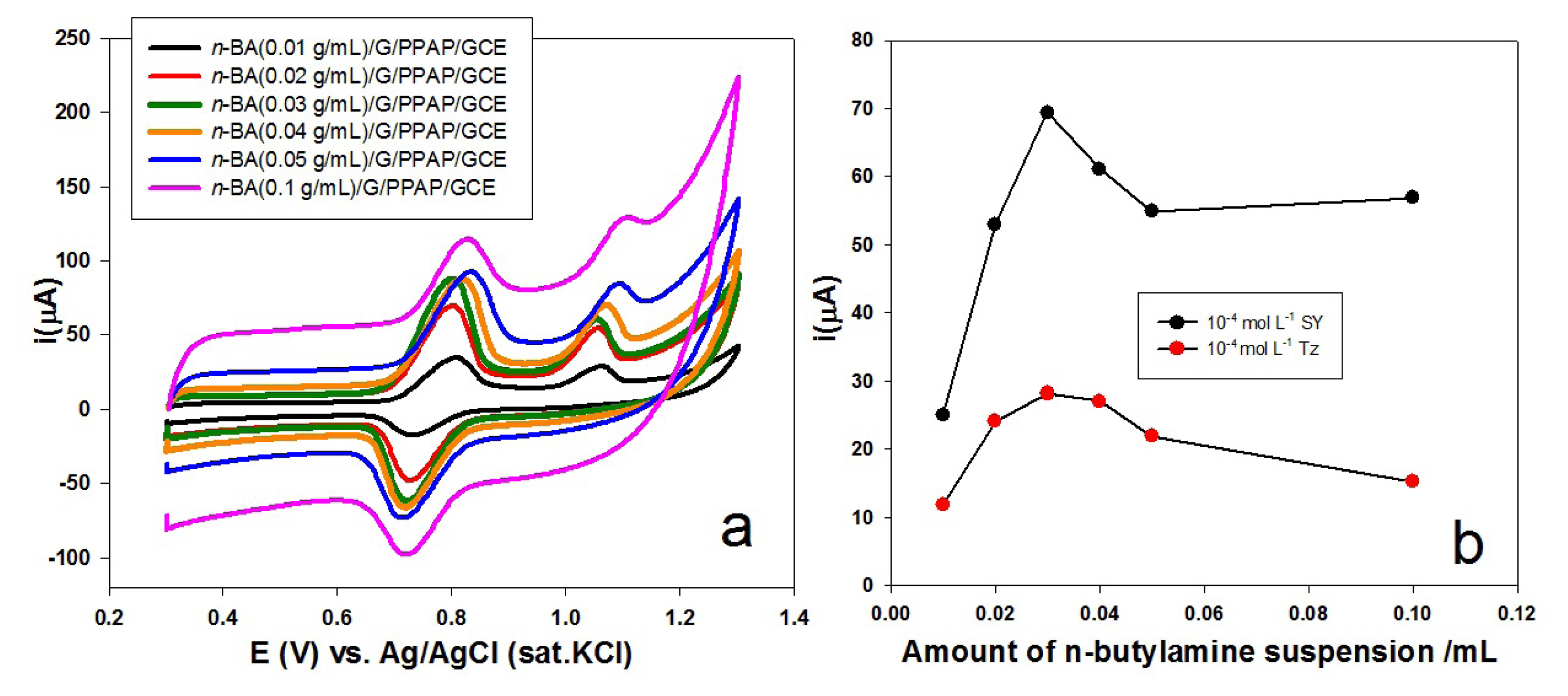

Supplement: Figure S5 — The cyclic voltammetric behavior of 1.0x10^−4^ mol L−1 SY and Tz prepared in different amounts of n-BA/G/PPAP/GC electrodes and related current quantity graph [file tjc-49-01-103s5.tif]
